# Nestin in the epididymis is expressed in vascular wall cells and is regulated during postnatal development and in case of testosterone deficiency

**DOI:** 10.1371/journal.pone.0194585

**Published:** 2018-06-06

**Authors:** Ansgar N. Reckmann, Claudia U. M. Tomczyk, Michail S. Davidoff, Tatyana V. Michurina, Stefan Arnhold, Dieter Müller, Andrea Mietens, Ralf Middendorff

**Affiliations:** 1 Institute of Anatomy and Cell Biology, Justus-Liebig-University Giessen, Giessen, Germany; 2 Institute of Anatomy and Experimental Morphology, University Medical Center Hamburg-Eppendorf, Hamburg, Germany; 3 Cold Spring Harbor Laboratory, Cold Spring Harbor, NY, United States of America; 4 Center for Developmental Genetics and Department of Anesthesiology, Stony Brook University, Stony Brook, NY, United States of America; 5 Moscow Institute of Physics and Technology, Moscow, Russia; 6 Institute of Veterinary Anatomy, Histology and Embryology, Justus-Liebig-University Giessen, Giessen, Germany; Universite Clermont Auvergne, FRANCE

## Abstract

Vascular smooth muscle cells (SMCs), distinguished by the expression of the neuronal stem cell marker nestin, may represent stem cell-like progenitor cells in various organs including the testis. We investigated epididymal tissues of adult nestin-GFP mice, rats after Leydig cell depletion via ethane dimethane sulfonate (EDS), rats and mice during postnatal development and human tissues. By use of Clarity, a histochemical method to illustrate a three-dimensional picture, we could demonstrate nestin-GFP positive cells within the vascular network. We localized nestin in the epididymis in proliferating vascular SMCs by colocalization with both smooth muscle actin and PCNA, and it was distinct from CD31-positive endothelial cells. The same nestin localization was found in the human epididymis. However, nestin was not found in SMCs of the epididymal duct. Nestin expression is high during postnatal development of mouse and rat and down-regulated towards adulthood when testosterone levels increase. Nestin increases dramatically in rats after Leydig cell ablation with EDS and subsequently low testosterone levels. Interestingly, during this period, the expression of androgen receptor in the epididymis is low and increases until nestin reaches normal levels of adulthood. Here we show that nestin, a common marker for neuronal stem cells, is also expressed in the vasculature of the epididymis. Our results give new insights into the yet underestimated role of proliferating nestin-expressing vascular SMCs during postnatal development and repair of the epididymis.

## Introduction

Nestin, a class VI intermediate filament protein, was first described in neuronal stem cells and emerged as a marker for these cells [1, 2]. Meanwhile, nestin is also found in other tissue-specific progenitor cells [1]. Nestin expression has been reported in different organs, especially during development and in adult organs associated with conditions of repair [3–5], or in cases of neoplasms and neovascularization [6–10].

Nestin has been localized to vascular walls [6, 8, 11–15]. Previously, it was suggested that adult vascular walls are completely differentiated and that circulating progenitor cells/ bone marrow-derived vascular progenitor cells exist for their repair [16, 17]. Recent results, however, describe additional progenitor cells residing in the vascular walls [6, 18–21]. Further studies have reported progenitor cells in the adventitia of adult blood vessels that express nestin [6] and are able to differentiate into other cells [6, 22].

Multipotent vascular stem cells have also been described as resident in the media of vessels [23]. In this context, studies reveal nestin expression in vascular smooth muscle cells (SMCs) and pericytes [11–13, 24]. In the testis, nestin-expressing vascular SMCs and pericytes could be identified as the progenitors of testosterone-producing Leydig cells [24] by use of the ethane dimethane sulphonate (EDS) model. A single injection of the cytotoxic compound EDS into adult rats eliminates the existing Leydig cells in the testis (with a subsequent decrease of testosterone levels) that is followed by a synchronized regeneration of Leydig cells imitating pubertal development [24, 25].

The expression of nestin in immature endothelial cells is also reported [15]. Nestin expression was suggested to occur in endothelial progenitor cells in the context of vascularisation, e.g. during the embryonic period [26, 27], during periodical organization of the uterus [28] and during tumour angiogenesis [6–10] Thus, nestin seems to be a marker for special cells in all layers of vessels that are not terminally differentiated and have a potential for proliferation.

The epididymis, localized on the dorsal side of the testis, consists of a single coiled duct that ensures transport, maturation and storage of spermatozoa initially released from the testis. Within the epididymis, three major regions are distinguished: head (caput), body (corpus) and tail (cauda). The epididymal duct is composed of the inner epithelial cells and the surrounding smooth muscle cell layer. During postnatal development, the epididymal duct grows and becomes coiled, connective tissue septa develop and divide the epididymis into different segments [29]. In parallel, the epididymal vasculature also grows to follow the development of the epididymal duct and ensure vascularization of the enlarging organ.

Data on the role of nestin in the epididymis are completely lacking. Here, we describe expression, localization as well as hormone- and development-dependent changes of nestin expression in rodents and man.

## Materials and methods

### Animals and tissues

Transgenic nestin-GFP mice were developed in the G. Enikolopov lab. An 8.7-kb fragment was purified by electrophoresis through the agarose gel and used for the pronuclear injections of the fertilized oocytes from C57BL/6 x Balb/cBy hybrid mice [30]. Mice express GFP under the control of the nestin gene promotor, enhanced by a transcriptional enhancer that resides in the second intron of the gene. After sedation with (400 μl of 15%) chlorylhydrate mice were perfused transcardially with 4% paraformaldehyde (PFA) in PBS [30]. PFA-fixed frozen tissues (n = 6) of these mice were provided for the study.

Tissues of C57BL/6N mice (Charles River Laboratories, Sulzbach, Germany) were used for assessment of the epididymis during postnatal development at postnatal day (d) d1, d4, d7, d10, d20 and d25 (n = 3 each). Mice were sacrificed by either decapitation (d1-d4) or by inhalation of 4–5% (v/v) isoflurane (d7-25). These tissues served for the real-time PCR analyses. All animal care and procedures followed the guidelines of the German Animal Welfare act, and use of the tissue was approved by the office for animal welfare of the Justus-Liebig-University Giessen (numbers A38/2011_V54-19c2015(1)GI20/23 and A29/2009_V54-19c20/15cGI20/23). Tissues of postnatal (d5) and adult (w12) male Wistar rats (n = 10 each), housed in the animal facilities of Justus-Liebig-University Giessen (number 527_A2), were used for immunohistochemical experiments, Western blot and real-time PCR analyses. Rats were sedated with 5% (v/v) isoflurane and killed by cervical dislocation.

For immunohistochemical and Western blot analyses of EDS-exposed animals, tissues from adult male Wistar rats (n = 10 each) that had received single i.p. injections (75 mg EDS/kg) were used. The rats had been killed 1–48 days after EDS injection. For assessment of mitotic cells, EDS-treated animals had received i.p. injections of BrdU (150 mg/kg body weight, dissolved in 0.09 M NaOH) 2 h before sacrifice [24]. Tissues were dissected immediately after decapitation of the animals and were either frozen in liquid nitrogen or fixed in Bouin's fluid [24]. The rats were used according to government principles regarding the care and use of animals with permission (G8151/591-00.33) of the local regulatory authority (Hamburg, Germany).

Human epididymal tissue (PFA-fixed and frozen; Bouin-fixed and paraffin-embedded) originated from patients undergoing epidido-orchiectomy for testicular cancer (n = 8) (permission by the Ethical Review Committee of the Ärztekammer Hamburg, Germany, OB.98; Sep 16, 1998).

### Immunostaining

Immunohistochemistry was performed on frozen (10 μm sections) and paraffin-embedded (5–6 μm sections) tissues from mouse, rat and man. Sections were incubated with the following antibodies: biotin-conjugated rat anti-mouse CD31 (clone MEC 13.3; BD Pharmingen, Heidelberg, Germany; dilution 1:50) and mouse anti-smooth muscle actin (SMA) (clone 1A4; Sigma-Aldrich, St. Louis, USA; 1:500) for our mouse samples only, mouse anti-proliferating cell nuclear antigen (PCNA) (clone EPR3821; Abcam, Cambridge, United Kingdom; 1:100) for all rodent samples, mouse anti-SMA FITC (clone 1A4; Sigma-Aldrich; 1:500) for rat and human samples, monoclonal mouse anti-nestin (clone R401; Chemicon, Schwalbach, Germany; 1:100) for rat samples only, mouse anti-nestin (Santa Cruz, Heidelberg, Germany; 1:50) for human samples only. For paraffin sections, the Envision kit (DAKO, Hamburg, Germany) was used for visualization according to the manufacturer`s instructions. Secondary antibodies used for frozen mouse, rat and human sections were Cy3 goat anti-mouse IgG (Jackson Immuno Research; 1:500). Cy3 streptavidin conjugate (Jackson Immuno Research Dianova, Hamburg, Germany; 1:500) was used after treatment with biotin-conjugated rat anti-mouse CD31 (see above). Negative controls in the absence of primary antibodies were performed for each immunostaining. Cell nuclei of frozen sections were additionally stained with 4',6-diamidini-2-phenyl-indole “DAPI” (Sigma, Munich, Germany).

For Clarity experiments nestin-GFP mouse tissue of approximately 1 mm^3^ was treated as described before [11]. Documentation was performed on a Zeiss LSM 710 Confocal Laser Scanning Microscope. Z-stacks were captured every 1.5–5 μm and reconstructed in ImageJ using the command “3D projects” allowing interpolation and contrast auto-enhancement if necessary.

### Protein preparation and immunoblotting

Separation of protein preparations by SDS-PAGE under reducing conditions and transfer of proteins to nitrocellulose membranes were performed as described before [24, 31]. After blots were stained with Ponceau S for loading control (Sigma-Aldrich) and non-specific binding sites blocked with non-fat dry milk, they were exposed to primary antibodies: mouse anti-nestin for rat samples (clone Rat 401: Millipore; 1:500), mouse anti-vinculin (clone hVIN-1: Sigma-Aldrich;1:30,000), mouse anti-β-actin (clone AC-15; Sigma-Aldrich; 1:20,000), rabbit anti-androgen receptor (clone EPR1535(2): Abcam, 1:1000). Goat anti-rabbit or anti-mouse IgG (Pierce, Rockford, IL, USA; 1:2000), linked to peroxidase, served as secondary antibodies. Immunoreactive bands were visualized by enhanced chemiluminescence.

### RNA isolation

RNA was extracted with the RNeasy Mini kit 50 (Qiagen, Hilden; Germany). Fresh native mouse and rat epididymal tissues were immediately homogenized in a ball mill with RLT-buffer and ß-mercaptoethanol. After centrifugation for 3 min (9520 xg) the supernatant was diluted in 600 μl ethanol 70% (v/v) and eluted by centrifugation through the RNeasy spin column. After two following washing steps the isolated RNA was dissolved in 30μl nuclease-free water. RNA was quantified using the Nanodrop 2000c (Thermo Scientific, Darmstadt, Germany).

Undesirable DNA products were removed by digestion with DNase I (Invitrogen, Karlsruhe, Germany). cDNA was synthesized in the Mastercycler Gradient (Eppendorf, Hamburg, Germany) and was performed with 1000ng/μl RNA according to the protocol of the iscript cDNA synthesis kit (Invitrogen).

### Quantitative real-time PCR (qPCR)

Quantitative real-time PCR was performed by using the iCycler IQ PCR system (Bio-Rad, Munich, Germany) and the SYBR-Green Mastermix (Invitrogen). Intron spanning primer pairs for nestin and the housekeeping genes RPS18 and β-actin were purchased from Eurofins (Hamburg, Germany). Primer sequences were: Nestin (152 bp mouse): Forward 5´- AGGCTGAGAACTCTCGCTTG—3´, Reverse 5´- TGAGAAGGATGTTGGGCTGA—3´. RPS 18 (167 bp mouse): Forward 5´- TGGTGTTGAGGAAAGCAGACAT—3´, Reverse 5´- GAACCTGGCTGTACTTCCCATC—3´. Nestin (214 bp rat): Forward 5´- GTAGACCCTTGGGTTAGAGGC—3´, Reverse 5´- TGGGCAATTCAAGGATCCCC—3´. β-actin (238 bp rat): Forward 5´- GCCATGTACGTAGCCATCCA—3´, Reverse 5´- GCACGATTTCCCTCTCAGCT—3´.

### Statistics

Relative expression of nestin during mouse postnatal development was analyzed using log-linear regression. The requirement of normal distribution of the residuals was met by logarithmic transformation of the data. Nestin expression in d5 vs. adult rat epididymis was compared by using an unpaired two-tailed t-test.

## Results

### Expression of nestin in the epididymis using the nestin-GFP mouse model

**Fig 1:**
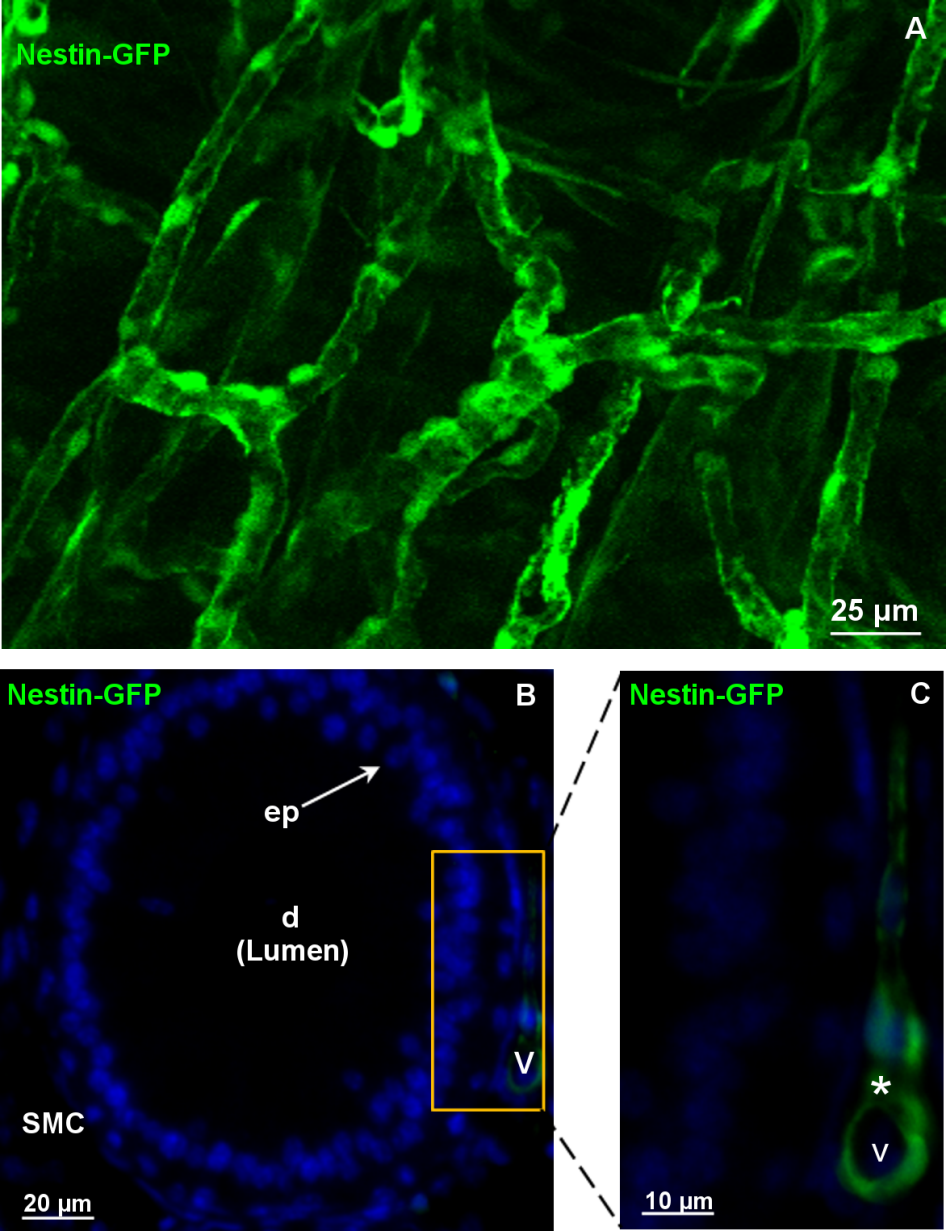
Localization of nestin-GFP in the vasculature of the murine epididymis. A: 3D-morphological analyses (depth 150μm): Nestin-GFP-positive cells are visible in the vasculature of the nestin-GFP mouse epididymis. B: Unlike blood vessels (v), epithelial cells (ep) and surrounding smooth muscle cells (SMCs) of the epididymal duct (d) are nestin-negative, DAPI (blue) labels the nuclei. C (B in a higher magnification): nestin-positive cells (asterisks) in the vasculature of the caput epididymidis.

The Clarity approach localized nestin to the vasculature and its three-dimensional distribution within the vascular network (Fig 1A). In another sample of the caput region, additional DAPI-staining revealed that only blood vessels were nestin-GFP positive (S1 Fig) different from the epithelial cells and the surrounding SMCs of the epididymal duct (Fig 1B and 1C).

**Fig 2:**
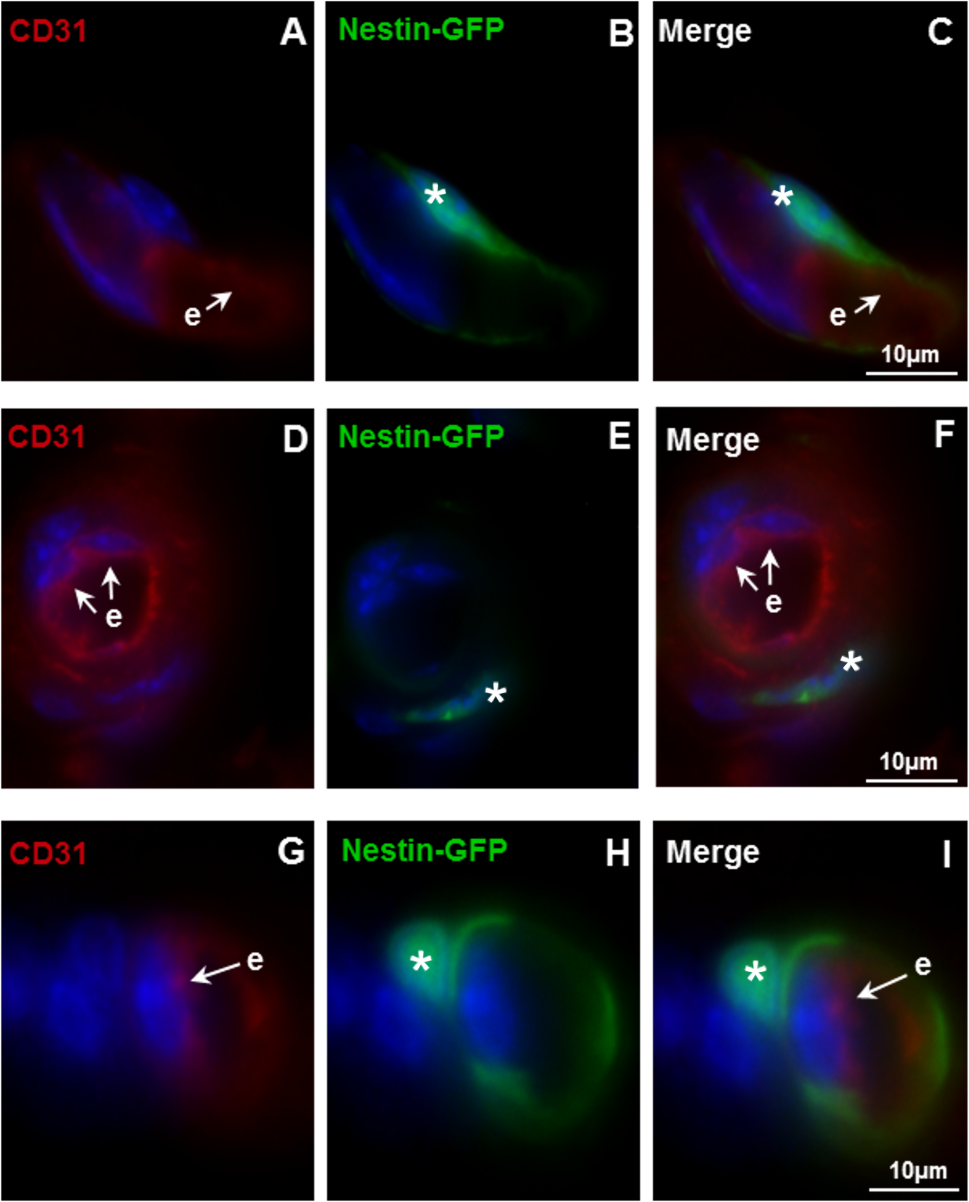
No colocalization of nestin-GFP and the endothelial cell marker CD31 in blood vessels. A-C,D-F,G-I: In vessels of the caput epididymidis., nestin-GFP is not colocalized with CD31 (red). DAPI (blue) labels the nuclei. A,D,G: CD31-positive endothelial cells (e). B,E,H: Nestin-GFP-positive cells (asterisks) in the vascular walls of the nestin-GFP mouse epididymis. C,F,I: Merged images.

**Fig 3:**
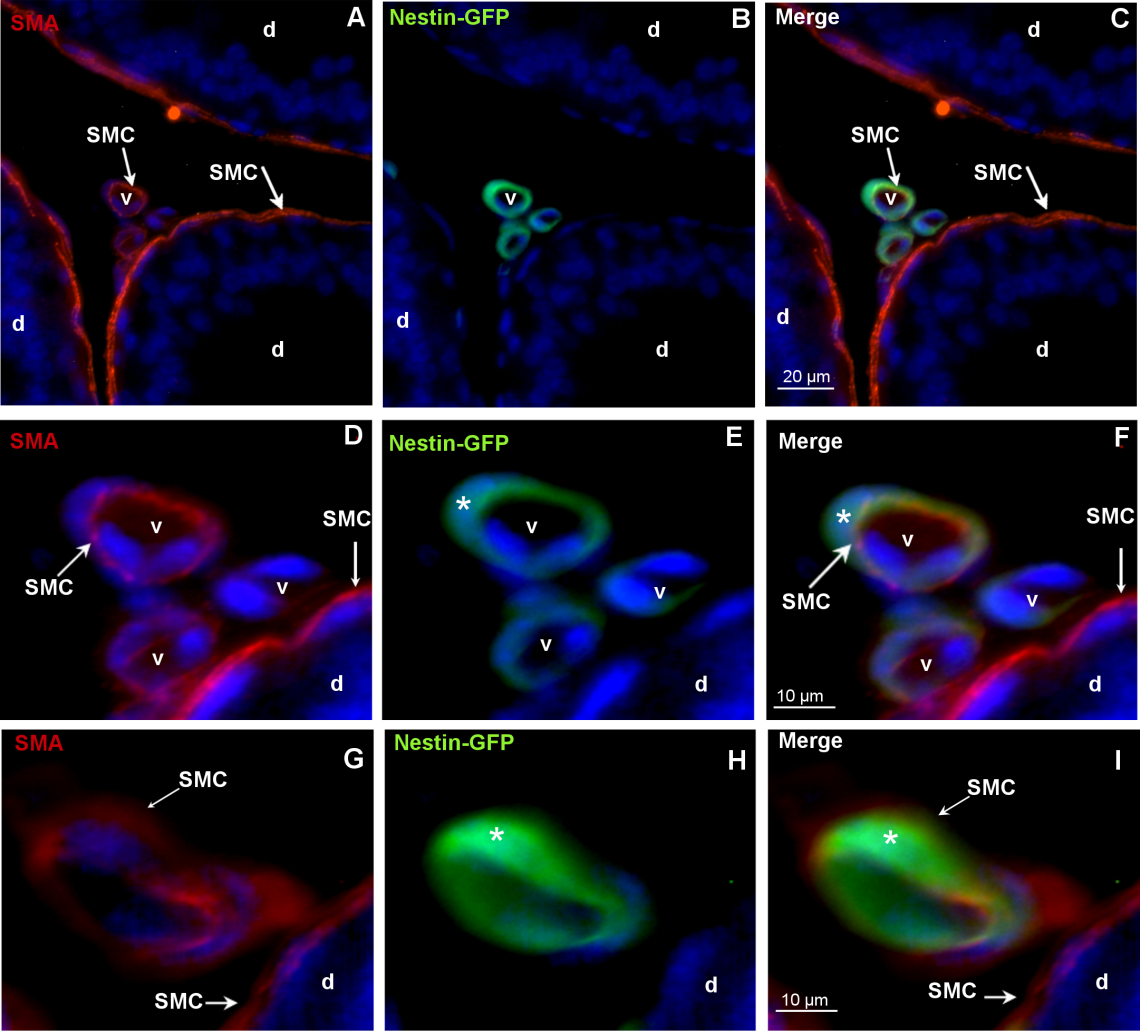
Colocalization of nestin-GFP and the SMC marker SMA in blood vessels. **A-F, G-I**: Different vessels (v) of the nestin-GFP mouse. The epididymis (caput) shows nestin-GFP-positive/SMA-positive SMCs. SMCs are indicated. DAPI (blue) labels the nuclei. **A,C,D,F,G,I:** SMA-positive SMCs of the vessels (v) and of the epididymal duct (d). **D-F:** Higher magnification of the vessels shown in A-C. **C,F,I**: Nestin-GFP-positive (asterisks) SMCs in vessels (v).

To clarify which cell type of the vessel expresses nestin in this mouse model, immunostainings with the endothelial marker CD31 and the SMC-specific marker SMA were performed. As shown here in the caput epididymidis (Fig 2C, 2F and 2I), no colocalization of CD31 and nestin-GFP was observed. The endothelial cells stained with CD31 indicated the innermost layer of the vessels and were clearly separated from the surrounding nestin-GFP-expressing cells (Fig 2A–2C, 2D–2F, 2G–2I). In addition to nestin expression in the cytoplasm, additional nestin-GFP was localized to cell nuclei by co-staining with DAPI (Fig 2B, 2C, 2E, 2F, 2H and 2I) [30]. SMA-positive SMCs were visible in the epididymal duct as well as in vessels (Fig 3A, 3C, 3D, 3F, 3G and 3I). Only in the vasculature SMCs were nestin-GFP-positive. Epithelial cells of the epididymal duct were negative both for SMA and nestin-GFP (Fig 3A–3C). Thus, nestin-GFP expression in the epididymis was exclusively detected in vascular SMCs (Fig 3C, 3F and 3I).

### Correlation of nestin expression with the proliferation marker PCNA

**Fig 4:**
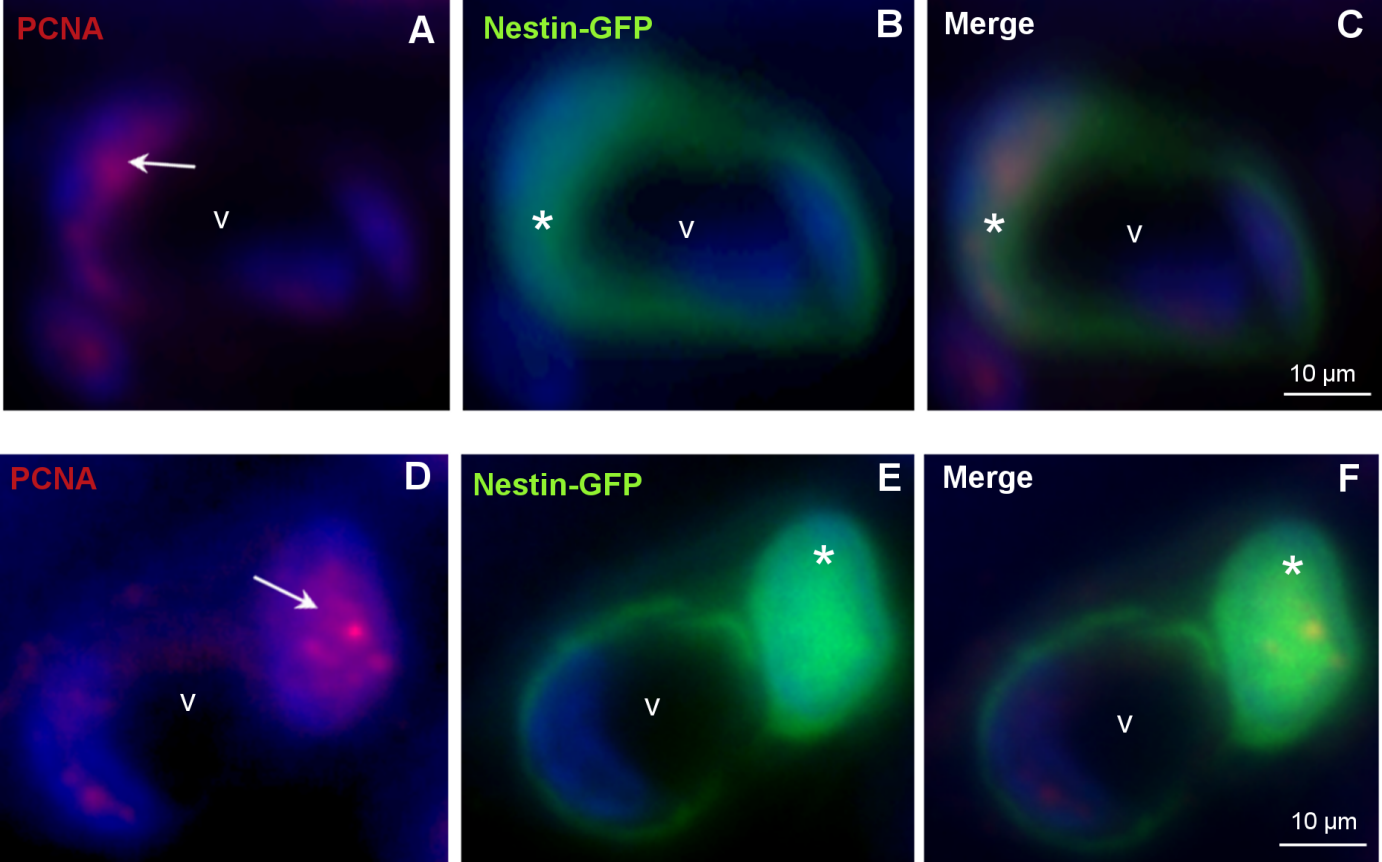
Colocalization of nestin-GFP and the proliferation marker PCNA. **A-C, D-F**: Different vessels (v) of the nestin-GFP mouse epididymis (caput) with nestin-GFP-positive / PCNA-positive cells. DAPI (blue) labels the nuclei. **A,D:** PCNA-positive cells (red) in the vascular wall (arrows). **B,E:** Nestin-GFP-positive cells (asterisks). **C,F:** Merged images.

To examine whether nestin-GFP-positive SMCs were proliferating cells, antibodies against the proliferation marker PCNA were used. A distinct staining for PCNA in the nuclei of the nestin-GFP positive cells was observed (Fig 4C and 4F). As expected some nuclei of endothelial cells also showed a positive signal for PCNA.

### Expression of nestin during postnatal murine epididymal development

**Fig 5:**
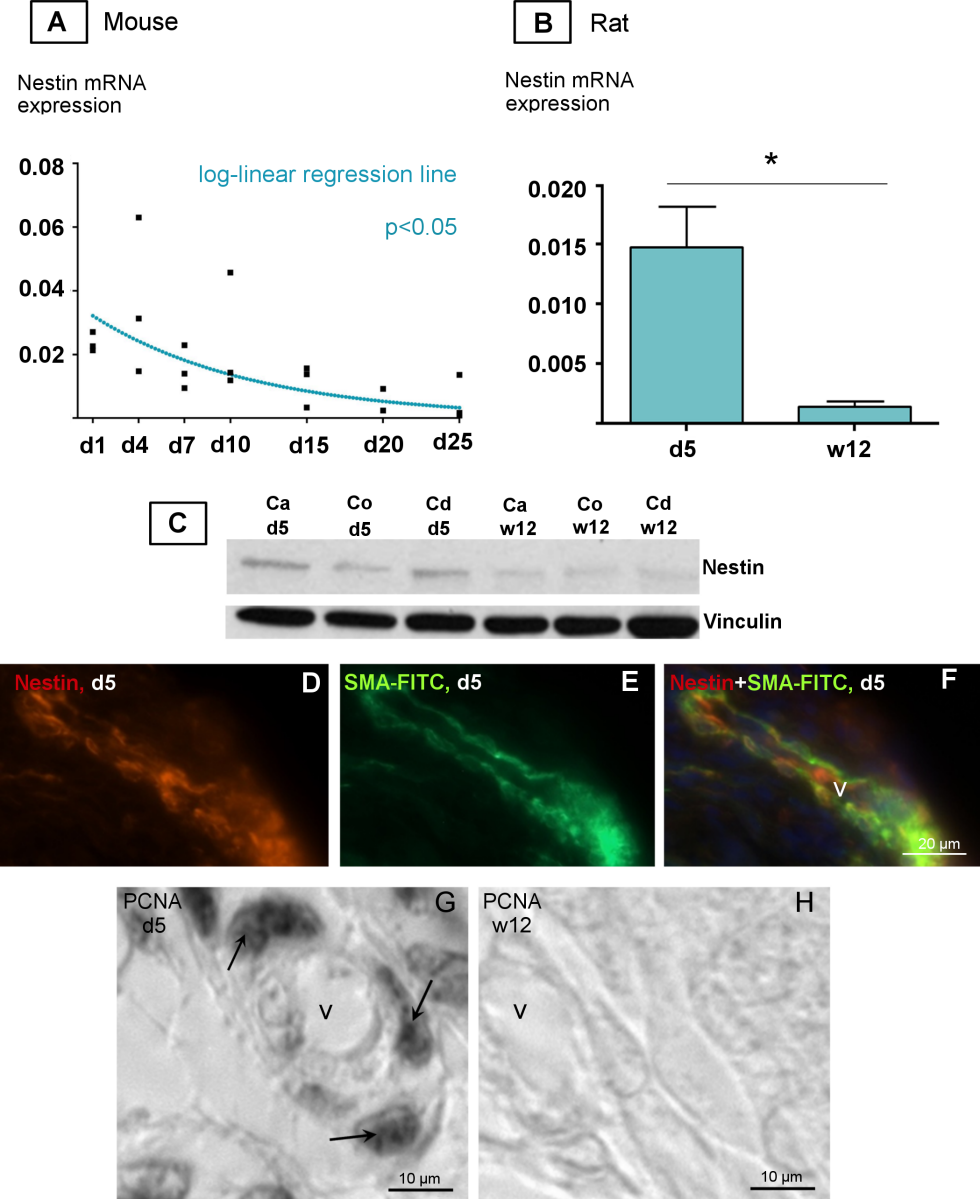
Nestin expression during postnatal development of the epididymis. **A-B:** qPCR analysis of nestin mRNA expression in the epididymis during postnatal development of mouse (A) and rat (B) (n = 3, each). **A:** relative nestin mRNA expression in the epididymis of the postnatal mouse decreases between d1-d25 as shown by log-linear regression (p≤0.05). Regression line is shown in blue. Housekeeping gene: RPS18. **B:** relative nestin mRNA expression in the epididymis of the postnatal rat (d5, w12). The error bars indicate SEM. *P<0.01 indicates significant differences. Housekeeping gene: β-actin. **C:** Western Blot analysis shows increased protein expression of nestin in the anatomical regions of postnatal epididymis, caput (Ca), corpus (Co) and cauda (Cd) in the 5 day-old compared with the 12w-old rat (20μg protein per sample). Vinculin was used as loading control. **D-F:** Immunofluorescence staining of the vasculature in the caput epididymidis of postnatal rats (d5) using antibodies against nestin (red) and SMA FITC (green). **D:** Nestin-positive cells in the vascular wall **E:** SMA FITC-positive vascular cells. **F:** Merged images. **G,H:** PCNA staining of paraffin embedded tissue. **G:** PCNA is highly expressed in the vasculature of the epididymis (arrows) on postnatal d5. **H:** No PCNA-positive cells are visible in the vasculature of the epididymis of the 12w-old rat.

The results clearly showed that nestin expression in mouse at the beginning of postnatal development was high and significantly (p<0.05) decreased towards d25 as shown by log-linear regression (Fig 5A, S2 Fig). Experiments with rat epididymal RNA preparations revealed significantly higher amount of nestin mRNA in the postnatal rat (d5) than in the adult rat (w12) (p<0.01) (Fig 5B). Western blotting also showed a high amount of nestin expression at the protein level in postnatal rats compared with that in adult rats. The higher nestin expression in postnatal rats was visible in all parts of the epididymis (caput, corpus, cauda) (Fig 5C). Immunostainings with anti-nestin and anti-SMA-FITC showed that nestin and SMA were also colocalized in epididymal vessels on d5 in the postnatal rat. This approach confirmed nestin localization by a different method in addition to the use of nestin-GFP transgene mouse tissue (Fig 5D–5F). Correlating to the high nestin expression at the beginning of postnatal development (d5) (Fig 5B and 5C), we also observed numerous vascular SMCs stained by the proliferation marker PCNA in the d5 rat (Fig 5G). In the adult rat (w12), however, PCNA-positive SMCs in the vasculature were barely detectable (Fig 5H).

### Nestin expression in the rat epididymis during testosterone deficiency after EDS treatment

**Fig 6:**
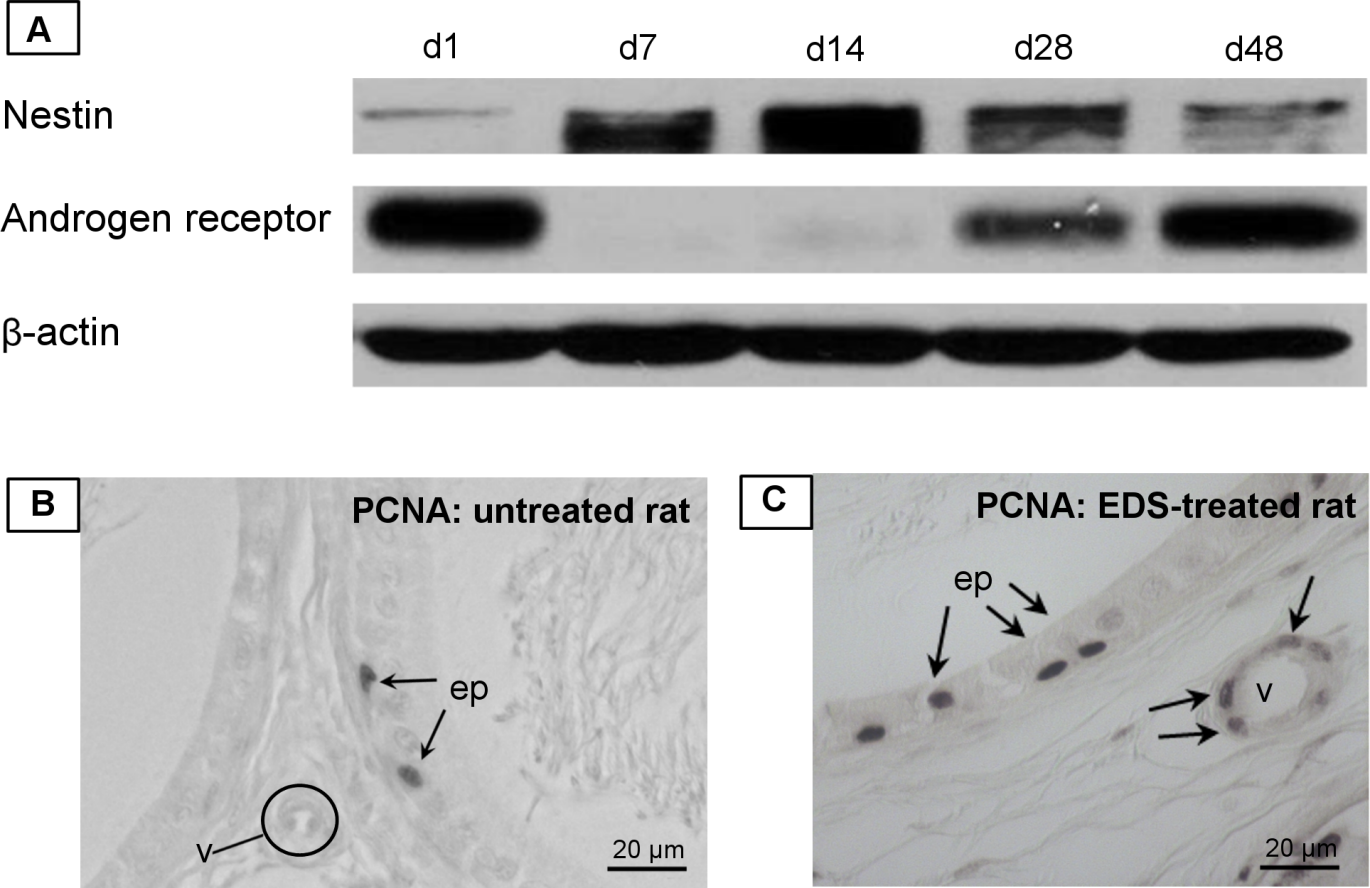
Epididymis after depletion of Leydig cells by EDS treatment. **A:** Western Blot analysis after EDS (ethane dimethane sulphonate) treatment in rats; on d7 and d14 after EDS a distinct increase of nestin expression is visible. On d28 and d48 nestin expression is lower again. The androgen receptor (AR) was barely detectable on d7 and d14. β-actin was used as loading control. **B,C:** Immunohistochemical staining of the rat epididymis (cauda) with the proliferation marker PCNA. **B:** PCNA-positive epithelial cells (ep) of the epididymal duct serve as an internal positive control. In a section of an untreated rat PCNA is only visible in epithelial cells (ep) but not in the vasculature (v). **C:** PCNA-positive cells are visible in a vessel (v) and in epithelial cells on d2 after EDS.

Western blot analysis revealed the highest increase of nestin expression in the epididymis (Fig 6A) on d7 and d14 after EDS treatment when Leydig cells are nearly completely absent [24]. On d48 after EDS treatment, when regeneration of Leydig cells in the testis is completed [25], epididymal nestin expression again decreased slightly (Fig 6A). However, in the same cytosolic protein fraction the expression of the androgen receptor (AR) in the epididymis behaved inversely and AR could barely be detected on d7 and d14. Interestingly, when nestin decreased, there was again an increase of AR expression in the epididymis (d28, d48) (Fig 6A). Comparing EDS-treated and untreated rats for proliferation markers PCNA (Fig 6B and 6C), we observed a relatively high number of PCNA-positive SMCs in blood vessels of EDS-treated rats different to control (Fig 6B).

### Nestin expression in the human epididymis

**Fig 7:**
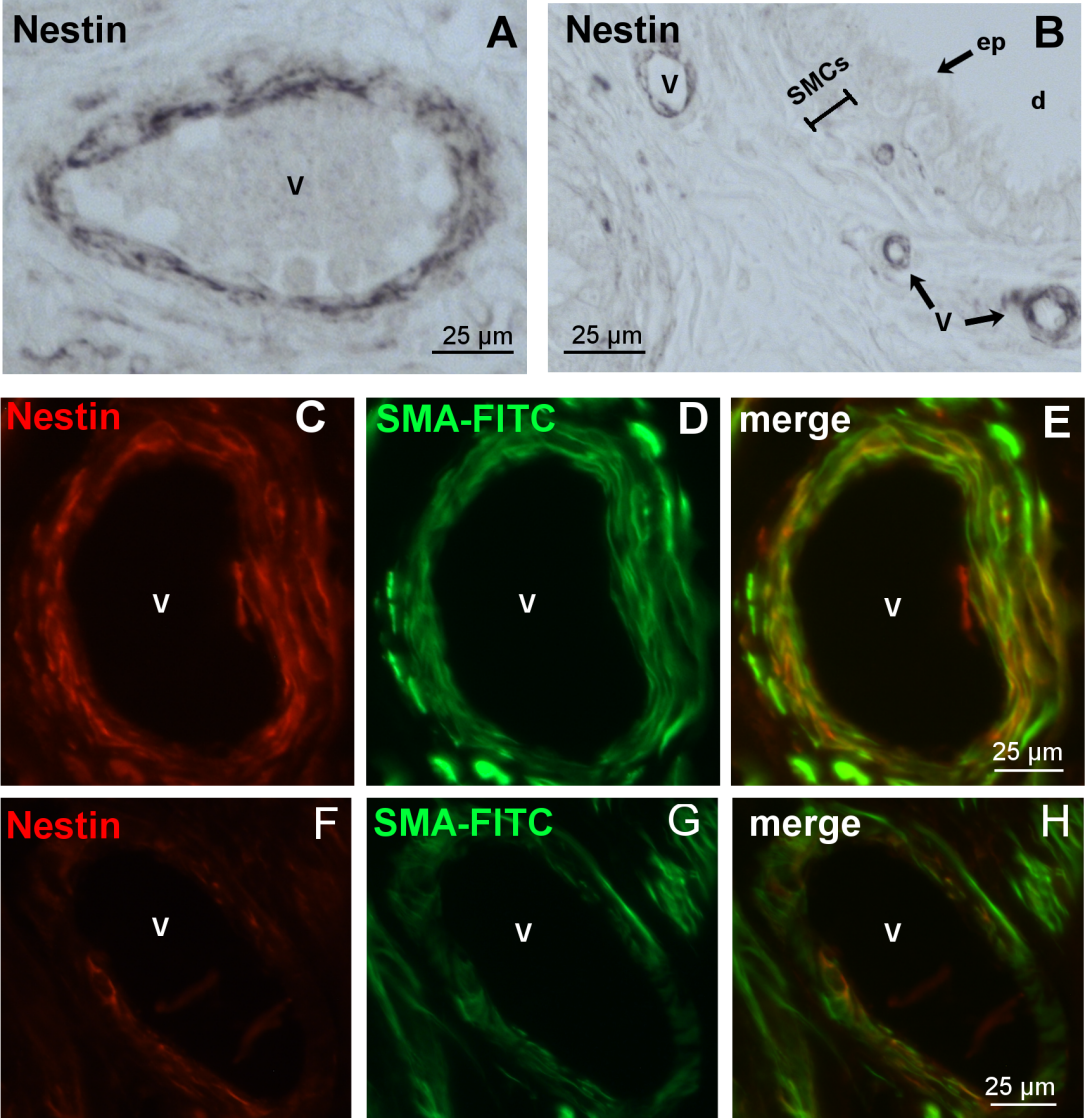
Nestin expression in vessels of the human epididymis. **A,B:** Paraffin sections (cauda) with nestin-positive cells in the blood vessels (v), but not in SMCs and epithelial cells (ep) of the epididymal duct (d). **C-E, F-H:** Immunofluorescence staining of two different vessels with antibodies against nestin (red) and SMA FITC (green). **E,H:** Merged image. It indicates the localization of nestin in vascular SMCs.

In human tissue we also succeeded in identifying nestin-stained vascular walls (Fig 7A and 7B). The staining was restricted to vascular SMCs and was absent from the epididymal duct SMC layer and the epithelium (Fig 7B). Nestin co-localization with SMA in the vascular walls confirmed the expression of nestin in vascular SMCs (Fig 7E and 7H).

## Discussion

In this study we describe the intermediate filament nestin for the first time in the epididymis. We could clearly localize nestin in the vascular SMCs unlike the SMCs belonging to the epididymal duct. We additionally showed increased nestin expression during postnatal development of the murine epididymis and during low testosterone levels after Leydig cell ablation by EDS treatment in rats.

### Nestin is expressed in vascular SMCs

Nestin expression could be identified in the vascular walls of the epididymis and unequivocally located to vascular SMCs by using nestin-GFP mouse tissue and immuno-staining for nestin, the endothelial cell marker CD31 and the SMC-specific marker SMA. Although nestin expression has been shown in vascular walls, the exact localization is still a matter of debate. It has been reported that vascular SMCs express nestin [11–13, 24], but nestin expression has also been described in endothelial cells, e.g. in proliferating endothelial progenitor cells [15], as precursors of endothelial cells in the lymph node [32] and in developing vessels after myocardial infarction [33]. However, in differentiated endothelial cells nestin expression has only been detected sporadically [8, 15, 28].

These differences in the literature about the exact localization of nestin in the different cell types and layers within the vessel wall could be explained by the grade of the development of the vessel. During embryonic development, vasculogenesis with de novo formation of vascular channels from endothelial progenitor cells predominantly occurs [34]. Thus, it is possible that in embryonic tissue nestin is expressed in endothelial progenitor cells. During postnatal and adult life angiogenesis is predominant with new vessels being generated from pre-existing blood vessels [34]. It is therefore conceivable that in the postnatal and adult phase nestin would also be detected in other vascular wall cells such as SMCs.

Remarkably, nestin expression was predominantly detected in smaller vessels with scattered SMCs as can be appreciated from the Clarity preparations. Presumably, some nestin-positive cells in small vessels represent capillary pericytes. From the expression of different pericyte markers [35], the variability and complexity of their origin, development and function [36], the identification of pericytes is difficult at the level of light microscopy. Thus, nestin-positive pericytes could have been occasionally incorrectly considered as endothelial cells in some previous reports. Nestin expression in pericytes has also been reported in other studies [24, 37, 38]. Pericytes, the perivascular cells of capillaries, have been repeatedly described as cells with stem cell characteristics [38] and the potential to differentiate [39, 40].

Interestingly, in rodent and human tissue nestin expression was only observed in the vascular walls but not in the contractile cells of the epididymal duct, comparable to the testis and lung, where nestin-positive cells represent a subpopulation of SMCs and pericytes of the vasculature, whereas SMCs of seminiferous tubules [24] and of the bronchial system [11], respectively, were nestin-negative.

### Nestin-expressing cells are proliferating cells

Since nestin is a marker for stem cells involved in the processes of development [41, 42] and regeneration, it was of interest to observe the proliferating characteristics of nestin-positive cells in the epididymis. Staining with PCNA revealed colocalization with nestin thus confirming that nestin-positive cells of the epididymal vasculature exhibit the potential for proliferation. Nestin expression in proliferating cells labeled by PCNA has also been reported in other studies, e.g. in progenitor cells in the forebrain of zebrafish [43] and in the glomeruli of the kidney [44]. Nestin-positive vessels also show more PCNA-labelled cells than nestin-negative vessels [7].

### High nestin expression during postnatal development of the epididymis with subsequent downregulation

We observed increased nestin expression during postnatal development of the epididymis in rat and mouse with highest expression around d4/d5 and a decrease during further development. It is well known that the postnatal period in the epididymis is of special importance for the development of the different cell types and the regional differences [45]. The maximum increase in weight of the epididymis as a surrogate for proliferation of cells during development is observed between postnatal days 25–30 [46]. Interestingly, highest nestin expression was seen earlier, namely on postnatal day 4, and thus could be a first sign of the following massive weight gain of the epididymis. This finding is comparable to nestin expression in a hypoxia-model of the lung, where nestin was identified as a very early marker of remodeling, preceding the proliferation of cells [11].

This observation of high nestin expression in the postnatal epididymis is similar to that in other organs that show increased nestin expression during postnatal development such as the testis and lung [11, 24]. Similarly, glial and neuronal progenitor cells in postnatal sympathetic ganglia are characterized by high nestin expression with a subsequent downregulation [47]. Additional immature and progenitor cells of embryonic and fetal tissue have been found to express nestin [1], e.g. developing podocytes of the kidney [48], developing cardiomyocytes [49], developing skeletal muscle cells [50], oval cells in developing liver [51] and epithelial cells of developing pancreas [27].

### Increased expression of nestin coincides with reduced levels of the androgen receptor in the epididymis after EDS treatment

We could observe a coincidence of clearly increased expression of nestin and reduced expression of the AR in the epididymis on days 7 and 14 after EDS treatment, when testosterone levels are extremely low [52]. When testosterone levels raised again in this model (day 25 after the EDS injection) [52], the expression of nestin decreased and the AR showed the same level as on day 1. So far, there is no information about an androgen responsive element in the nestin gene.

Decreased AR after EDS treatment has been described in the nuclei of Sertoli cells in the rat testis [49]. During the first week after EDS administration even a total loss of AR immunostaining was described with subsequent recovery of AR in these cells [53]. Whereas AR mRNA levels in whole testis did not change 5 days after EDS treatment, in the epididymis and prostate AR mRNA levels increased [54]. At the protein level, however, Blok et al. [54] showed reduced AR levels in the testis after EDS administration corresponding to our epididymis data. AR protein in the epididymis was not investigated by these authors [54]. Other early investigations have also described the influence of androgen on the expression of its receptor [55, 56]. In our study, there was no evidence that downregulation of AR in the EDS model was due to intracellular translocation of the receptor. This was shown by comparing cytosolic with total protein (including additional nuclear and membrane proteins) of the epididymis where we observed a decreased AR in the absence of testosterone.

To the best of our knowledge the observed inverse expression pattern of nestin and AR have not been described so far. On d7 and d14 after Leydig cell ablation (when testosterone levels are very low), we observed high levels of nestin in the epididymis. Consistently, high nestin expression was also found in the testis 7 and 14 days after EDS treatment with a low number of immunoreactive Leydig cells [24], but a direct correlation between nestin and AR in testis or in other organs has not been described so far.

## Conclusions

In summary, we revealed the localization of nestin in the epididymis in vascular SMCs of mouse, rat and human tissue, but not in SMCs of the epididymal duct. We could demonstrate the proliferating characteristics of nestin-expressing cells and the distinct increase of nestin during postnatal development and as a response to injury by EDS treatment. Thus, it is possible to describe nestin as an indicator for dynamic processes during development and regeneration and repair.

## Supporting information

S1 Fig Epididymal section of the nestin-GFP mouse at lower magnification. Nestin-GFP-positive cells are visible in the vasculature of the nestin-GFP mouse epididymis. DAPI (blue) labels the nuclei.
(TIF)

S2 Fig qPCR analyses for nestin-expression during postnatal development of the mouse epididymis. **A:** raw data set for relative nestin-mRNA expression in the mouse epididymis. Housekeeping gene: RPS18. **B:** Residuals for the log-transformed relative nestin-expression were proven to be Gaussian as visualized by the normal Q-Q-plot.
(DOCX)
