# Supplementary figures and images for: Nestin in the epididymis is expressed in vascular wall cells and is regulated during postnatal development and in case of testosterone deficiency
Source: PLoS One. 2018 Jun 6;13(6):e0194585. doi: 10.1371/journal.pone.0194585 (PMC5991371; doi:10.1371/journal.pone.0194585)

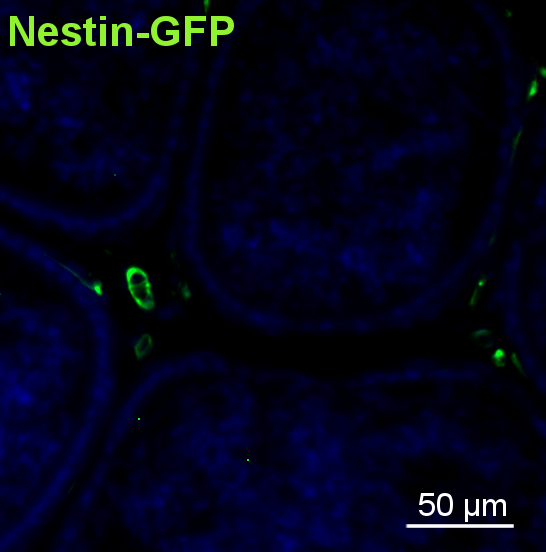

Supplement: S1 Fig — Nestin-GFP-positive cells are visible in the vasculature of the nestin-GFP mouse epididymis. DAPI (blue) labels the nuclei. (TIF) [file pone.0194585.s001.tif]
