# Supplementary material for: Nestin in the epididymis is expressed in vascular wall cells and is regulated during postnatal development and in case of testosterone deficiency
Source: PLoS One. 2018 Jun 6;13(6):e0194585. doi: 10.1371/journal.pone.0194585 (PMC5991371; doi:10.1371/journal.pone.0194585)
Supplement: S2 Fig — A: raw data set for relative nestin-mRNA expression in the mouse epididymis. Housekeeping gene: RPS18. B: Residuals for the log-transformed relative nestin-expression were proven to be Gaussian as visualized by the normal Q-Q-plot. (DOCX) [file pone.0194585.s002.docx]

| d1 | 0,0219 |
| --- | --- |
|  | 0,0268 |
|  | 0,0213 |
| d4 | 0,0303 |
|  | 0,0622 |
|  | 0,0147 |
| d7 | 0,0229 |
|  | 0,0140 |
|  | 0,0094 |
| d10 | 0,0444 |
|  | 0,0123 |
|  | 0,0119 |
| d15 | 0,0136 |
|  | 0,0154 |
|  | 0,0033 |
| d20 | 0,0089 |
|  | 0,0089 |
|  | 0,0023 |
| d25 | 0,0017 |
|  | 0,0137 |
|  | 0,0008 |

| Age (days) | Relative nestin-expression |
| --- | --- |

B

A


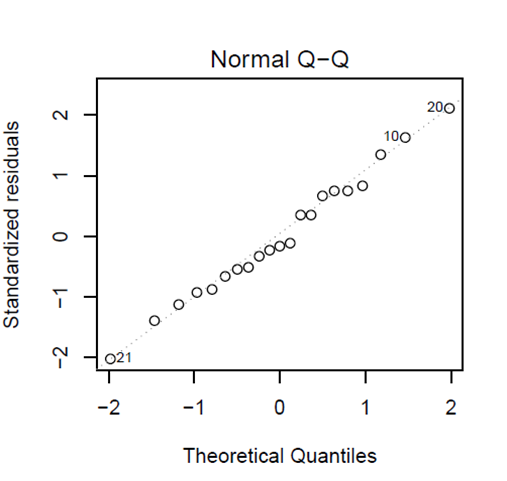


**S2 Fig: qPCR analyses for nestin-expression during postnatal development of the mouse epididymis.**

**A:** Raw data set for relative nestin-mRNA expression in the mouse epididymis. Housekeeping gene: RPS18. **B:** Residuals for the log-transformed relative nestin-expression were proven to be Gaussian as visualized by the normal Q-Q-plot.
